# Determinants of antibiotic prescribing for upper respiratory tract infections in an emergency department with good primary care access: a qualitative analysis

**DOI:** 10.1017/S095026881800331X

**Published:** 2019-03-08

**Authors:** Y. Y. Chan, M. A. Bin Ibrahim, C. M. Wong, C. K. Ooi, A. Chow

**Affiliations:** 1 Department of Clinical Epidemiology, Office of Clinical Epidemiology, Analytics, and Knowledge, Tan Tock Seng Hospital, Singapore, Singapore; 2 Department of Emergency Medicine, Tan Tock Seng Hospital, Singapore, Singapore

**Keywords:** Antibiotic prescribing, emergency department, physician, qualitative, upper respiratory tract infections

## Abstract

Upper respiratory tract infections (URTIs) account for substantial attendances at emergency departments (EDs). There is a need to elucidate determinants of antibiotic prescribing in time-strapped EDs – popular choices for primary care despite highly accessible primary care clinics. Semi-structured in-depth interviews were conducted with purposively sampled physicians (*n* = 9) in an adult ED in Singapore. All interviews were analysed using thematic analysis and further interpreted using the Social Ecological Model to explain prescribing determinants. Themes included: (1) reliance on clinical knowledge and judgement, (2) patient-related factors, (3) patient–physician relationship factors, (4) perceived practice norms, (5) policies and treatment guidelines and (6) patient education and awareness. The physicians relied strongly on their clinical knowledge and judgement in managing URTI cases and seldom interfered with their peers’ clinical decisions. Despite departmental norms of not prescribing antibiotics for URTIs, physicians would prescribe antibiotics when faced with uncertainty in patients’ diagnoses, treating immunocompromised or older patients with comorbidities, and for patients demanding antibiotics, especially under time constraints. Participants had a preference for antibiotic prescribing guidelines based on local epidemiology, but viewed hospital policies on prescribing as a hindrance to clinical judgement. Participants highlighted the need for more public education and awareness on the appropriate use of antibiotics and management of URTIs. Organisational practice norms strongly influenced antibiotic prescribing decisions by physicians, who can be swayed by time pressures and patient demands. Clinical decision support tools, hospital guidelines and patient education targeting at individual, interpersonal and community levels could reduce unnecessary antibiotic use.

## Introduction

Upper respiratory tract infections (URTIs) or the common cold are illnesses presenting with acute inflammation of the nasal or pharyngeal mucosa but without other specifically defined respiratory conditions such as streptococcal tonsillitis, pneumonia and asthma [1]. URTIs are one of the most cited reasons for antibiotic use [1]. However, antibiotic therapy for URTIs, which are predominantly of viral aetiology, has not been shown to provide any clinical efficacy [1]. Excessive and unnecessary use of antibiotics for the treatment of URTIs is prevalent in the USA where over 30% of adults presenting with uncomplicated URTIs and 75% with acute bronchitis were prescribed antibiotics [2–4]. From 2001 to 2010, 126 million (12.2%) emergency department (ED) visits in the USA were for acute respiratory tract infections, with almost half (47.9%) of the antibiotic-inappropriate infections being administered antibiotics [5]. Adults had the highest rate of antibiotic use for antibiotic-inappropriate acute respiratory infections (URTIs, influenza and viral pneumonia), with 500 per 1000 visits by adults aged 20–64 years and 666 per 1000 visits by those aged 65 years or older from 2009–2010 [5]. In the UK, despite clinical guidelines advising against the use of antibiotics for URTIs [6], about 47% were prescribed antibiotics in general practices [7].

Inappropriate use of antibiotics subject patients to unnecessary adverse effects of antibiotics and drives antimicrobial resistance [8]. Antibiotic misuse is the most important preventable cause of antimicrobial resistance in both hospital and ambulatory settings [9–11]. Although antimicrobial stewardship has been widely promoted in hospitals [12, 13], it has focused largely on inpatient settings and has excluded EDs [14, 15]. The US Centers for Disease Control and Prevention has recently highlighted the importance of antibiotic stewardship in EDs [16].

Globally, research in EDs has shown that besides patients’ clinical factors, clinical decision-making by ED physicians are challenged by non-clinical factors such as frequent overcrowding [17, 18], rapid turnover of patients and staff, a shift-based workplace [19] and concerns of failure to diagnose and treat [20]. Physicians prescribe antibiotics to avoid uncertainty and risks of deterioration of the patient's health status [21, 22], when pressed for time [20, 21, 23, 24], when they perceived patients to be of a lower socio-economic status [24, 25], when they perceived that patients expected antibiotics [25, 26] or to increase patient satisfaction [26, 27].

In Singapore, URTI is the leading condition managed at primary care clinics, accounting for one-quarter of consultations [28]. Despite the presence of highly accessible primary care clinics in the community, URTIs accounted for a substantial proportion of attendances at EDs and were associated with frequent ED attendances [29]. Approximately 24% of adult patients with URTIs in EDs were inappropriately prescribed antibiotics [30]. Despite this substantial proportion of inappropriate antibiotic prescribing there is a lack of qualitative studies in countries with highly accessible primary care clinics that can help provide an in-depth and comprehensive understanding of the determinants of antibiotic prescribing in the ED. To date, only one study has attempted to examine factors associated with antibiotic decision-making by ED physicians using qualitative methods [19]. The study was conducted in the USA where EDs served as safety nets for vulnerable populations with limited access to primary care [31]. The findings might not be applicable in countries such as Canada, France, UK, Saudi Arabia, Australia and Singapore, where there is good primary care coverage [32].

Qualitative methods are increasingly used as a complement to quantitative methods and can help uncover the antibiotic prescribing influences for URTI that are unique to EDs in areas with highly accessible primary care clinics and guide the development of context-appropriate interventions to reduce inappropriate antibiotic use [19, 27, 33].

We, therefore, conducted an exploratory qualitative study at the ED of a large adult tertiary-care hospital in Singapore which is a developed country with advanced emergency services [34] and highly accessible primary care clinics [35], to understand the determinants influencing antibiotic prescribing decisions among ED physicians who attend to adults presenting with URTI.

## Methods

### Study design

In-depth interviews (IDIs) were conducted with purposively sampled physicians working in the busiest adult ED in Singapore. The ED at the 1600-bed Tan Tock Seng Hospital attended to an average of 450 patients daily. Physicians were the only healthcare providers who prescribed medications for patients. A semi-structured interview guide (Table 1) was developed and piloted with two senior physicians for comprehensiveness and acceptability for use. The interview guide explored participants’ knowledge and behaviours towards URTI treatment, perceived norms of managing URTIs, and views on antibiotic resistance. Demographic data of participants such as age, gender, country of basic medical training, years of clinical practice and current position were also collected.

**Table 1.** Interview guide questions

***1. Behaviour and attitude towards the treatment of URTI***
How would you usually treat URTI cases? What are your reasons for prescribing antibiotics? What are your reasons for not prescribing antibiotics? Under what circumstances would you prescribe antibiotics for the treatment of URTI? Are there any non-clinical factors that may play a part in your decision to prescribe/not prescribe antibiotics? Are there situations when you feel uncertain as to whether or not to prescribe antibiotics for URTI? What makes you say that? How do you handle the feelings of uncertainty or ambiguity? Are there situations when you feel pressured to prescribe antibiotics? Can you elaborate? What happened in the end? Can you share with me how you learnt of these treatment methods or approaches?
***2. Perceived norms regarding the treatment guidelines of URTI at ED***
What do you think is the common practice of treating URTI at this ED? How do you handle situations when your prescribing decisions are not in alignment with your peers? (If guidelines were mentioned, ask:) Can you tell me more about the guidelines that you mentioned? What is your opinion regarding the use of these guidelines? How do you think these guidelines can be improved?
***3. Views on antibiotic resistance for URTI cases***
I would like to understand your views regarding antibiotic resistance for URTI cases. What is your opinion about antibiotic resistance? How do you think issues of antibiotic resistance can be addressed? Do you feel that the concern for antibiotic resistance influences whether you prescribe antibiotics for URTI? With regard to antibiotic resistance, are there other suggestions or strategies that you would like to see introduced?

### Data collection

IDIs were conducted between March and May 2016. Prior to the commencement of the IDIs, a list of physicians working in the ED was obtained from the hospital's Medical Affairs Office. After consultation with the study team's co-investigator who is based in the ED, it was revealed that junior physicians were usually assigned to attend to URTI cases in the ED. As such, the study team purposively sent out email invitations to resident physicians, senior residents and medical officers to participate in the study. This enabled the study team to interview information-rich participants who were able to provide in-depth insights into the factors that influenced antibiotic-prescribing decisions for URTI cases in the ED. The email invitations emphasised voluntary participation in the study. This was also reiterated by interviewers prior to the commencement of each interview. Participants gave written consent to participate in the study and for their interviews to be audio-recorded. Participants were not offered any incentives or reimbursements but were provided with some snacks and drinks during the IDIs. Two researchers trained in qualitative research conducted the IDIs. Each interview lasted approximately 90 minutes.

In addition to purposive sampling of participants who were able to provide in-depth insights into the phenomenon of interest, we ensured the adequacy of the study's sample size using the guiding principle of data saturation. Data saturation is defined as the point when no additional themes emerge, and data begin to repeat as data collection proceeds [36, 37]. The sample size for the study was guided by Namey *et al*. [36], who found that the median number of interviews required to reach a threshold of 80% saturation was eight (range: 5–11 interviews). Hennink *et al*. [37] further examined 25 IDIs to assess the concept of data saturation and found that code saturation or the identification of the range of thematic issues was reached at nine interviews. We initially conducted seven IDIs and analysed these for themes and subthemes. Thereafter, we conducted two additional interviews to assess for thematic saturation. Analysis of data from additional interviews found no new emerging themes.

### Analysis

Audio recordings were transcribed verbatim and reviewed for accuracy by a third study team member. After reading the transcripts, a preliminary codebook was developed and study team members independently coded a randomly selected transcript. Inter-rater reliability was ascertained by comparing and resolving any discrepancies in coding. Study team members repeated the process to arrive at a final codebook which was used to code the remaining transcripts. Codes were organised using QSR International's NVivo 10 software. These were subsequently summarised and analysed using applied thematic analysis [38]. Descriptive analyses were used to summarise the demographic data of the participants.

## Results

### Participant demographics

Nine physicians were included in the study, with a good distribution of age, experience and place of medical training. The demographics of the participants are shown in Table 2.

**Table 2.** Demographics of study participants

Demographic characteristics	No. of participants (*n* = 9)
Age (years)	
21–29	3
30–39	3
40–49	3
Gender	
Male	4
Female	5
Basic medical training	
Singapore	3
UK	1
Philippines	3
Myanmar	1
Ireland	1
Years of practice	
1–4	1
5–9	5
⩾10	3
Years of practice in ED	
1–4	4
5–9	3
⩾10	2
Current position	
Senior resident	5
Senior resident physician	2
Medical officer	2

### Determinants that influence antibiotic prescribing

**Fig. 1.**
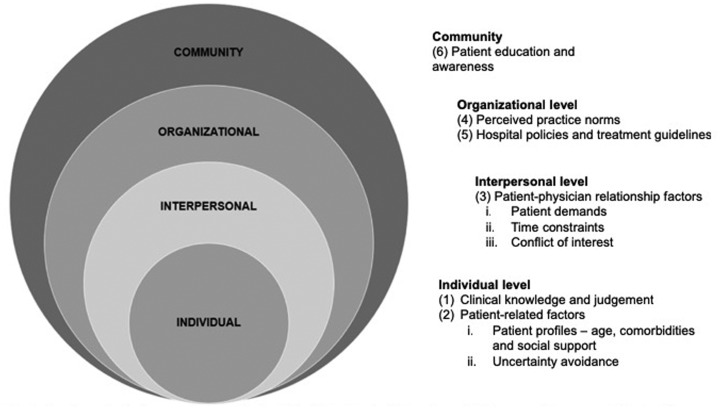
A socio-ecological approach to understand the determinants influencing antibiotic prescribing among ED physicians.

Six key themes emerged as determinants influencing the clinical management of URTI, namely (1) clinical knowledge and judgement, (2) patient-related factors, (3) patient–physician relationship factors, (4) perceived practice norms, (5) policies and treatment guidelines and (6) patient education and awareness. These were further interpreted using the Social Ecological Model (SEM) as a conceptual framework. To understand why and when ED physicians prescribe antibiotics, it is necessary to understand the interactions between ED physicians and the environments they work in. The SEM allows the identification of social and organisational leverage points that can be targeted in interventions to help reduce inappropriate antibiotic prescribing [39]. Figure 1 shows the overview of the socio-ecological approach in understanding the determinants influencing antibiotic prescribing.

### Individual level

#### Clinical knowledge and judgement

At the individual level, physicians utilised their clinical knowledge and judgement when providing care for URTI patients. When deciding between symptomatic treatment and antibiotics, all participants relied strongly on their clinical knowledge and work experiences. They would prescribe symptomatic treatment for uncomplicated URTIs as they believed antibiotics were unnecessary and might instead cause adverse effects.

For simple URTI, if it's the first time I see the patient…that means the patient didn't see any doctor prior…I will give symptomatic relief for fever and cough. That would be like Paracetamol, cough suppressants, decongestants and lozenges. That's all. I'm not going to give antibiotics the first time because I believe it's viral. Participant 5

Additionally, all participants reported that they worked independently and were confident of their clinical decisions. They attributed their capabilities to prior training in practicing evidence-based medicine and guidance from senior physicians. Other major influences on prescribing decisions for URTIs were results of laboratory and radiologic investigations, and clinical practice guidelines.

*You see…don't be bothered by other people's practice. From what I've been through here, what I see with this patient and that patient…they probably have the same histories. But you may not actually know how fast this infection will progress in each patient. If I have to give* [*antibiotics*]*…I have to give. I won't be bothered by what my colleagues will say because in the end, I am actually looking after the patient. Participant 9*

Furthermore, antibiotic prescribing practices were influenced by patient-related factors such as age, comorbidities as well as avoiding uncertainty in diagnoses.

#### Patient-related factors

- Patient profiles – age, comorbidities and social support

Majority of the participants reported a lower threshold in prescribing antibiotics for elderly patients, especially those with comorbidities or were immunocompromised. The main reasons were to prevent any potential deterioration of the patient's illness or occurrence of secondary bacterial infections. The availability of social support for elderly patients was also taken into consideration.

So first thing…major factor is age. So if the patient is very elderly…I would say URTI is a diagnosis of exclusion. So I usually would investigate first by doing blood tests, x-rays to rule out lower respiratory tract infection and look at the clinical status of the patient. So the threshold for starting antibiotics would be lower for me in the older age groups. Simply because they deteriorate faster. Participant 7

- Uncertainty avoidance

Participants would also prescribe antibiotics when they were uncertain of diagnoses or for fear of not providing timely and appropriate treatment. These included patients with borderline blood test results or were persistently symptomatic after a course of antibiotics, particularly in elderly persons with comorbidities.

*Because sometimes as a primary care* [*doctor*]*… you may have the fear that he may never return again. There's a lot of ‘what ifs’ that you ask yourself as a primary care. What if this is the only time I see this patient? And what if he has something serious? What if I'm missing something? Participant 4*

### Interpersonal level

Prescribing practices were also influenced by the dynamics of the patient–physician relationship such as patient demands and time constraints.

#### Patient–physician relationship

- Patient demands

Most participants reported previous experiences of pressure to prescribe antibiotics from demanding patients or their family members, who perceived that antibiotics would relieve their symptoms and cure URTIs.

Sometimes they demand for antibiotics. Usually I try to counsel them. These are usually the young, and well-educated kind. If they insist, I would usually give it to them but tell them to not to take it first…to like keep it on standby in case the fever persists for like another few days…. Participant 1

- Time constraints

Time constraints were cited as a critical factor in managing URTI patients. As such, the workload required effective time management to attend to all the patients within the work shift. A few participants shared the trade-offs between spending the time to educate patients on the appropriate use of antibiotics and acceding to demands to allow for more consultation time with other patients.

And of course we need to be realistic, time is also important. We're managing the whole queue. We're managing a lot of patients, and we can't spend an unlimited amount of time to deal with a difficult consult. We need to know…we need to decide on what is safe for the patient and what is a good balance. Participant 4

- Conflict of interest

Participants shared about educating and convincing patients that antibiotics were not necessary for URTIs of viral aetiology. However, they had to manage patient expectations and achieve patient satisfaction.

I make sure I educate them. That in the end…next time…maybe the antibiotics won't be useful for them when they need it. But if they still insist then I'll prescribe it to them. I have educated you and you can take it at your own risk. Participant 1

### Organisational level

Referring to the organisational and work culture within the ED, participants reported that they were accorded high levels of autonomy in providing care for URTI patients.

#### Perceived practice norms

All participants believed that symptomatic management was the standard of care for URTIs in the ED. However, patients were prescribed antibiotics when laboratory tests suggested a bacterial infection or as reserve treatment should symptoms worsen.

*This ED* [*referring to the ED in this hospital*] *is symptomatic* [*in terms of providing treatment for URTI*]. *I mean that's what we teach our residents and medical officers. Most of the time we just give them the Piriton or the Loratidine and you know the phlegm-stoppers, the cough-stoppers, the fever-stoppers. It's very symptomatic in management. Participant 6*

Furthermore, most participants seldom encountered misalignments in prescribing decisions with their peers or seniors. If decisions differed, three participants mentioned that they would question the senior physicians or their peers while one would defer to the senior physician's decision. When exceptional cases were flagged out, most participants felt these were useful case studies for learning.

I don't think people would interfere with your management of an URTI. I think they sort of try, if you're trying to bring it up…there may be something they want to learn also because obviously it's not just the normal run of the mill cases. Participant 2

Additionally, one participant observed that junior physicians were less confident than senior physicians in not prescribing antibiotics for URTI. He was concerned that junior physicians were more likely to succumb to external pressures and prescribe antibiotics unnecessarily.

*I don't know whether this is backed by evidence. Anecdotally, I find that a higher proportion of junior doctors feel less confident about not prescribing antibiotics. I've also observed some of the resident physicians who I would classify as more experienced physicians giving antibiotics more readily than the consultants or registrars. Participant* 4

#### Hospital policies and treatment guidelines

Participants reported no specific hospital policy on the management of URTI. Two participants pointed out that ED physicians were prudent in prescribing antibiotics, usually in the best interests of their patients. One participant expressed concerns that introducing hospital policies for antibiotic use in the ED could impede his work and hinder the exercise of appropriate clinical judgement.

*I wouldn't think that hospital policy would help me. In fact, it is a shackle because it prevents me from exercising my own judgment. I know some people may consider that as a hospital policy or departmental policy, so as not to give* [*antibiotics*] *for the first presentation or whatever. I think that is more problematic than useful, so I don't like that. Participant 4*

Some participants mentioned the existence of guidelines and resources for managing URTIs such as those from the American Family Physician Association, ‘UpToDate’ website, and the hospital's antibiotic stewardship guidelines. In contrast to hospital policies, participants expressed a strong preference for these guidelines as they could still exercise their clinical judgements. However, participants considered the scope of such guidelines to be too broad and believed that their clinical judgement was more valuable. Two participants acknowledged that the hospital's homegrown inpatient antibiotic computerised decision support system, ‘ARUS-C’ [40], might be useful for improving antibiotic prescribing in the ED setting, indicating preferences for prescribing guidelines based on local epidemiology.

*Usually when you start working here, the intranet* [*hospital's internal website*] *will provide you with access to ‘UpToDate’ and usually you can search for everything inside. So you will get the latest trends on what they recommend, what their randomised trials are showing, what were the previous results, what would be the percentage and what would be the wrong approach or wrong management. And if you want to know further details, you can click on their link. It is useful, but you still need to apply your clinical judgment obviously. You cannot say ‘Oh okay…the UpToDate website is like this…so I will just treat all my patients this way. Every patient is unique and it may not be the same as what is in the textbook or whatever.’ Participant 5*

### Community level

#### Patient education and awareness

In response to suggestions on ways to address issues related to antibiotic resistance, all participants felt that it was critical to reinforce the appropriate use of antibiotic therapy among primary care providers, increase patient education and awareness on managing URTIs, and to address misconceptions regarding the use of antibiotics to treat URTIs. Three participants mentioned the use of educational leaflets to inform patients and their families on the treatment and management of URTI, to empower self-management of symptoms.

Because… I mean most clinicians are quite aware of the problem of antibiotic resistance. But the public in my opinion quite ignorant about this. I mean you look at the health seeking habits of Singaporeans. It is really like you know…for URTI, they will see one doctor. If not better, they go to another doctor. You know…they ‘doctor shop’. So I mean continuity of care is one issue. The other thing also is antibiotic resistance. The clinicians may not know what the previous doctor prescribed. If they prescribed something else, then… you know for one URTI they can receive two or three courses of antibiotics… which is really…really bad. So I think a lot of these issues require public education. Participant 7

A summary table consisting of all themes, subthemes and corresponding quotes, which are interpreted according to the SEM, are provided as a Supplementary Table.

## Discussion

This study provided insights into the clinical and non-clinical determinants influencing antibiotic prescribing by ED physicians for adult URTIs in a country with highly accessible primary care clinics. The study also adopted a socio-ecological approach in understanding the determinants that interact to influence the ED physician's decision to prescribe antibiotics.

Integrating socio-behavioural sciences into the understanding of antibiotic prescribing practices can enable the effective planning and development of interventions to enhance clinical care for better patient outcomes [33]. This study revealed that antibiotic prescribing behaviours and practices among ED physicians were shaped by clinical and non-clinical determinants that spanned the interactive socio-ecological bands.

First, at the individual level, we observed that ED physicians had the autonomy in decision-making. Although the practice norms in the ED were for the symptomatic treatment of URTIs, physicians reported a lower threshold to prescribe antibiotics when faced with clinical factors such as older patients and comorbidities. Physicians were also uncertainty avoidant when presented with equivocal results from laboratory and radiologic investigations. These findings corroborated with the observations in other studies [19–21, 30]. Clinical decision support tools could address the uncertainties faced by the physician and improve antibiotic prescribing decisions in the ED [41]. At the interpersonal level, we observed that physicians tended to prescribe antibiotics for demanding patients and when constrained by time. Other studies similarly found that physicians were more likely to prescribe antibiotics if they believed that patients expected them [24], and that the fast-paced environment of ED encouraged unnecessary antibiotic use [19].

At the ED-specific organisational level, this study highlighted the deep-rooted culture of the ED of practicing evidence-based medicine. Guidance by senior physicians appeared to be a prominent factor for practice norms. Senior physicians were sources of information and role models in shaping one's approach towards antibiotic prescribing. Other studies have shown that supervisors and senior colleagues played a highly influential role in prescribing behaviours of hospital doctors in the early stages of their careers [15, 42]. Therefore, the provision of continuing education to senior physicians in the ED can enhance antibiotic prescribing practices in the ED [43]. We also noted that physicians’ perceived limited applicability of clinical practice guidelines and existing hospital clinical decision support systems on antibiotic prescribing for URTI, coupled with patients’ poor understanding on antibiotics use, could have contributed to over-prescribing in response to patient demands and time pressures. As physicians highly valued their clinical experience, implementation of any clinical guidelines in the hospital would necessarily have to be based on most up-to-date data on the local epidemiology of URTI and latest international evidence on the management of URTI.

Lastly, this study found that physician's decision to prescribe antibiotics was influenced at the community level by patient expectations for antibiotics in the treatment of URTI that were likely to have arisen from inadequate knowledge and misconceptions of antibiotics. Physicians felt that there was a critical need to educate the general population and patients on the proper use of antibiotics, and to create the awareness of antibiotic resistance. Patient education with a combination of messages providing information on self-management of symptoms and explanations on the inappropriateness of antibiotics for URTI could reduce antibiotic prescribing for URTI [44]. Three study participants suggested the distribution of educational leaflets to patients as they waited outside the consultation rooms. This could be incorporated into the workflow of the ED.

This study adds to the limited published literature on understanding the determinants of antibiotic prescribing in ED settings. Our observations corroborated with the influencing factors described by May *et al*. [19], although in the context of a different healthcare system where primary care was highly accessible. Notably, access and quality of care received outside the ED consult, which emerged as a theme in the study by May *et al*. [19], was not observed in our study. While the other themes observed by May *et al*. [19] were similar to ours, we had used the SEM to illustrate the interdependence and interactive effects of the individual-, interpersonal-, organisational- and community-level factors. A quantitative study involving all physicians practicing in the ED is underway for triangulation of findings, and the expansion of the study to involve all major adult EDs in Singapore is being planned to gain deeper insights into the cultural- and organisational-level factors at EDs.

The study has several strengths. Researchers who were not related to the ED conducted the interviews, providing observations that were unlikely to be biased. They were familiar with the research topic and had received extensive training in qualitative research data collection techniques. Additionally, researchers started each interview by building rapport with participants and reassuring them of the confidentiality and anonymity of data. As such, participants were observed to be forthcoming and candid with responses, particularly when describing the ED's practice values and norms, challenges faced and the workplace culture. Hence, the study's findings were likely to be authentic.

The study is limited to ED physicians managing adult URTI patients, and hence findings might not be generalisable to ED physicians attending to paediatric patients. Despite not being generalisable to such settings, the study provided detailed descriptions of the phenomena experienced by ED physicians in the Results section and Supplementary Table so that readers can assess the transferability and applicability of the study findings to similar contexts and situations [45]. Nonetheless, the study has provided important insights into antibiotic prescribing that might guide antimicrobial stewardship in adult EDs with good primary care networks.

## Conclusions

Organisational practice norms strongly influenced antibiotic prescribing decisions by physicians for adults presenting with URTI at the ED. Although physicians have a preference for evidence-based practice, they often prescribe antibiotics inappropriately when faced with time pressures and patient demands. Clinical decision support tools, hospital guidelines and patient education could be useful in reducing unnecessary antibiotic use.

## Supplementary material

For supplementary material accompanying this paper visit https://doi.org/10.1017/S095026881800331X. click here to view supplementary material
