# Supplementary material for: Determinants of antibiotic prescribing for upper respiratory tract infections in an emergency department with good primary care access: a qualitative analysis
Source: Epidemiol Infect. 2019 Mar 8;147:e111. doi: 10.1017/S095026881800331X (PMC6518493; doi:10.1017/S095026881800331X)
Supplement: Supplementary file 1 [file S095026881800331Xsup001.docx]

| **Supplementary Table 1**  Socio-ecological level of influence, corresponding themes and summary of corresponding quotes |
| --- |
| ***Individual level*** |
| **Clinical knowledge and judgment**   1. “For simple URTI, if it’s the first time I see the patient…that means the patient didn’t see any doctor prior…I will give symptomatic relief for fever and cough. That would be like Paracetamol, cough suppressants, decongestants and lozenges. That’s all. I’m not going to give antibiotics the first time because I believe it’s viral.” – Participant 5 |
| 1. “You see…don’t be bothered by other people’s practice. From what I’ve been through here, what I see with this patient and that patient…they probably have the same histories. But you may not actually know how fast this infection will progress in each patient. If I have to give [antibiotics]…I have to give. I won’t be bothered by what my colleagues will say because in the end, I am actually looking after the patient” – Participant 9 |
| 1. “I guess through here when you do postings in GM [general medicine] you see the sort of sicker side of it. You see the pneumonias and how it can actually deteriorate. And you learn that you know…the elderly people have a sort of lower threshold…more easy to deteriorate. And I guess you see also through trial and error, like you see patients re-attend because they didn’t get antibiotics or you see patients attend because they had an adverse reaction to the antibiotics that maybe they didn’t need. So then from that I think your clinical judgment is always like shifting, when you take these into account.” – Participant 2 |
| 1. “In the textbook they will say URTI, you do not need to give antibiotics. Unless there is say evidence like… total white blood counts raised, and findings from the chest x-ray. That’s the textbook saying right? But clinically sometimes, if they are treated partially… they won’t say anything about that in the textbook. And then some of the seniors will tell you, “oh okay you ask the patient how long they have had this”. This is the patient’s history. And then sometimes patients will tell you their sputum colour… like white sputum…no worries, yellow sputum is fine, but greenish and like rusty you might think about something’s wrong and that’s something’s important.” – Participant 5 |
| 1. “Most people assume that you know how to treat URTI. I think at least back in my days in medical school, they assume that you know how to treat URTI. Or it’s simple enough for you to treat on your own. Of course when you grow older, you realise [it] may not be as straightforward and you need a bit more finesse in certain cases. It’s a combination of being taught by seniors, reading guidelines…there are actually a lot of guidelines on this like, the AFP, the American Association of Family Physicians, there’s the published, those like those CENTORS guidelines that kind of thing.” – Participant 4 |
| 1. “I learnt everything here in this hospital, I would tell you that. Because I started working here in respiratory medicine. So I had very good teachers, I was very fortunate to have very good.” – Participant 6 |
| 1. “Ah well because I came from another country [Name of Country removed], so yeah… mainly from my clinical experience there and of course when I came here… of course with the guidance of the… our bosses.” – Participant 3 |
| 1. “Usually I would sort of try and compromise with them, I would tell them if I clinically think it’s not indicated, I would explain what we are doing for them, and explain why. I would show them the X-ray or I would show them the blood test and say this is the normal limits, your tests fall within the normal limits, you know…it’s very good news for you, I don’t think it’s anything serious going on and try and sort of turn that on their head because they are trying to sort of look for evidence that they are sick, in the sense. But you know, always try and say you know, it’s very good news! You’re not seriously ill!” – Participant 2 |
| 1. “We do things to help us make a decision, simple full blood count…simple chest X-ray…helps rule out a lot of things, but it is not complete.” – Participant 4 |
| ***Interpersonal level*** |
| **Patient-related factors** |
| 1. **Patient profiles – Age, comorbidities and social support** |
| 1. “So first thing…major factor is age. So if the patient is very elderly… I would say URTI is a diagnosis of exclusion. So I usually would investigate first by doing blood tests, x-rays to rule out lower respiratory tract infection and look at the clinical status of the patient. So the threshold for starting antibiotics would be lower for me in the older age groups. Simply because they deteriorate faste*r*.” - Participant 7 |
| 1. “It depends on how old they are, the elderly ones…I am more inclined to err on the side of caution and prescribe antibiotics.” – Participant 2 |
| 1. “Patients with clear URTI…who are significantly immunocompromised like patients undergoing chemo, patients undergoing or taking immune drugs, the rheumatology patients the whole lot of them there…I would have a lower threshold for giving them antibiotics.” – Participant 6 |
| 1. “Strep A or Strep B… these infections may likely need antibiotics but most of the time, if it’s actually viral in nature, I just actually give symptomatic treatment. But in cases where you are actually confounded with immunocompromised patients such as those ones with cancer, or whatever…they are on steroids, allergies, autoimmune disorders, we’ll probably have to start with antibiotics which I usually do. All those which I know are really already bacterial, I’ll will really start antibiotics, yup.” – Participant 9 |
| 1. “I suppose patient and family demands. For example, patients who frequently admit for similar kind of things, you seem to…you sort of get a sense that…you know you have ten admissions in the last one year for URTI. There must have been something which led them to being admitted. Is it because their family refused to take the patient home? Is it because the patient cannot care for himself? Is that patient at high risk of falling? Even when it’s URTI and sepsis etcetera. So I guess patient’s past history, patient’s social set up, whether the family can care for them at home etcetera.” – Participant 2 |
| 1. “I would say social factors are another important aspect of the care. So if the patient is elderly, quite frail…meaning the patient has a few comorbidities, but with very poor social support…and he refuses further investigations or whatever. Maybe he declined da blood test which may have been indicated or he declined a chest X-ray, if I was originally thinking of one…then sometimes I would consider giving antibiotics if I thought there may be some possibility of a lower respiratory tract infection, for example. So that’s one. But as juxtaposed to if he had very good social support, then even if there was some doubt, if the clinical diagnosis of URTI is like not more than 90% or 100%...there is some doubt but you think he is still quite well with good social support. Then I may actually hold off the antibiotics first. Because there’s somebody to look out for this patient and you know that when the patient turns ill, he would be brought back to a clinical facility for reassessment. So in those cases, I would probably be a bit less inclined to give antibiotics. Social support does play some role la. Yeah, in more in more for the elderly.” – Participant 4 |
| 1. **Uncertainty avoidance** |
| 1. “If I think that the diagnosis of URTI is not firmed…Sometimes if it’s like an older person that has like other comorbidities and… the cough is purulent, then I might still prescribe them with antibiotics.” – Participant 1 |
| 1. “Because sometimes as a primary care [doctor]… you may have the fear that he may never return again. There’s a lot of “what ifs” that you ask yourself as a primary care. What if this is the only time I see this patient? And what if he has something serious? What if I’m missing something?” – Participant 4 |
| 1. “Yeah, if they are having persistent fever and persistent symptoms. And you know if the blood test or the X-ray are equivocal. I mean if the blood test looks very viral… like those where the total whites are really low and platelets are low, then I would be much less keen to give them antibiotics. I would think that looks more like a viral picture on the full blood counts.” – Participant 1 |
| 1. “Uh…the elderly, with multiple comorbidities or a condition which may cause them to be in a sense immuno-compromised or relatively immunocompromised. Whether is it severe, poorly controlled diabetes, some kind of malignancy ongoing…that kind of thing. So those patients we always are very hyper vigilant because they do not present typically. They may not have fever, sometimes they may not have much dyspnoea…but you know they are doing poorly because they are not eating well, you know they are very dehydrated, even though they have URTI, sometimes those patients make you think twice whether you may be missing something...” – Participant 2 |
| 1. “So very often if the infection markers are a little bit elevated, even if they are like twelve, for example, they are not floridly twenty, thirty...In fact, those cases tend to be more ill and they are often admitted. But if like say just borderline elevated maybe twelve, with some neutrophilia, chest X-ray pending the report you think there is some haziness somewhere. Then I will clear cut give because there is a significant enough possibility this is a lower respiratory tract infection and there may be more benefit than harm in giving la. Yeah.” – Participant 2 |
| 1. “Well the usual of course is treating them symptomatically. Erm…we usually just give antibiotics if necessary but of course…if the patient already received antibiotics and the patient is not responding. Let’s say patient is already received antibiotics from the GP or from any doctor, and the patient is still clinically ill…not responding to the medications given to them, so we usually prescribe antibiotics.” – Participant 3 |
| **Patient-physician relationship** |
| 1. **Patient demands** |
| 1. “Sometimes they demand for antibiotics. Usually I try to counsel them. These are usually the young, and well-educated kind. If they insist, I would usually give it to them but tell them to not to take it first…to like keep it on standby in case the fever persists for like another few days...”– Participant 1 |
| 1. “Sometimes it’s patients’ expectations as well. Like you know, they will say, you know I went to the GP…he refused to give me antibiotics. Now I’ve come here because…that’s what I want.” – Participant 2 |
| 1. "A lot of it is about their expectation. Like they come to the ED even for minor sprains, expecting X-rays and things like that. And you know if you don’t do it, they say “oh you’re not a real doctor you know, maybe I should go to another hospital [name of hospital removed]”. And in a lot of the cases if truly that’s what the patient expects and that’s what they want, you know, you can tell them clinically I think you should be ok, but if really you want then… I will give you if I don’t think it’s actively going to harm you although it may not help.” – Participant 2 |
| 1. “It’s basically…judging your patient. If the patient is anxious, the patient is stubborn, has a pre-conceived expectation and idea that I’m coming to the emergency to get antibiotics, so I better be getting my antibiotics. So then I’ll just give them, I won’t sit there trying to argue with you and talk you out of it.” – Participant 6 |
| 1. **Time constraints** |
| 1. “So you think about the risk and benefits you know, if I stay here arguing with this patient and building up a worse doctor-patient relationship with them for the next thirty minutes, the opportunity costs of that is…I could have seen three other patients who genuinely need my help, and avoided a complaint letter to the Minister of Health, Minister of Manpower etcetera.” – Participant 2 |
| 1. “And of course we need to be realistic, time is also important. We’re managing the whole queue. We’re managing a lot of patients, and we can’t spend an unlimited amount of time to deal with a difficult consult. We need to know…we need to decide on what is safe for the patient and what is a good balance?” – Participant 4 |
| 1. **Conflict of interest** |
| 1. “I make sure I educate them. That in the end…next time…maybe the antibiotics won’t be useful for them when they need it. But if they still insist then I’ll prescribe it to them. I have educated you and you can take it at your own risk.” – Participant 1 |
| 1. “Usually I will explain to [the] patient. Your symptoms are like this and that. And then you might need to take the antibiotics. But this is the benefits if you take the antibiotics…this is the risks if you take the antibiotics. If patient say okay, I want to try the antibiotics. Okay fine... so I will prescribe the antibiotics. And then if like a straightaway case… not responding, concuss… I won’t ask the patient. If like very vague, if patient is young, I look through using my clinical judgment.” – Participant 5 |
| 1. “I guess you see it’s all about balance. So if you think the patient needs it, I wouldn’t won’t worry about the resistance if the patient clinically requires it. If I think the patient is more on the borderline, I would go with sort of my gut feel of the patient. Do you think they are going to need it? I suppose as in…I was brought up…as in I did medicine in the UK where they have the concept of delayed prescribing. So you will give them a prescription and say in forty-eight hours if your flu hasn’t gotten better, then start the antibiotics. I don’t think we do that as much here.” – Participant 2 |
| 1. *“*The decision is with the patient… I will explain to the patient again. I will give like antibiotics; you can have it even though it is likely viral. But there is a risk... like you might have an antibiotic induced diarrhoea or if you stop…you might have resistance.” – Participant 5 |
| ***Organizational level*** |
| **Perceived practice norms** |
| 1. “This ED [referring to the ED in this hospital] is symptomatic [in terms of providing treatment for URTI]. I mean that’s what we teach our residents and medical officers. Most of the time we just give them the Piriton or the Loratidine and you know the phlegm-stoppers, the cough-stoppers, the fever-stoppers. It’s very symptomatic in management.” Participant 6 |
| 1. “Okay… like if my colleague sees a patient before, and then patient comes back with same symptoms… and then I’m still going to see if there is a blood test or any evidence that I need to prescribe antibiotics. That’s number one… since we use evidence based medicine. If there is no evidence [to] show that we need antibiotics, I will still agree with my colleague. I am not going to give antibiotics. I am just going to assure patient that it still might be viral. So I am not going to give antibiotic.” – Participant 5 |
| 1. “So I think I believe I stand for most…based on my…not just observation but also my understanding of my peers, I find it that most of the more seniors ones are more confident in managing URTI appropriately and are aware that inappropriate use of antibiotics may actually do more harm than good or yeah and in fact not help the problem. I say so because I’ve seen many times when the seniors clear the cases for the juniors, we question what the juniors write, when they consider you know, they say keep in view antibiotics because of for example prolonged or projected course of a viral symptom. But there is no other evidence that will support the use of the antibiotic. Then the senior will be very steadfast and stand by the decision not to. So I’ve seen observed all these, I think most of my fellow peers practice quite similarly.” – Participant 4 |
| 1. “Well I guess most of the time we just manage our own cases ourselves, so doesn’t really matter, yeah. Usually my consultants won’t go and bother with my decisions about URTI patients, yeah most of the time…” – Participant 1 |
| 1. “I’m the one seeing the patient, the particular patient and every patient is different. I don’t think someone here will pressure you to compare notes or what because you’re not seeing the same patient. Yeah mostly for this URTIs, one doctor one patient…they can be discharged after usual consultation.” – Participant 8 |
| 1. “I suppose if I were to look at the throat and I saw that the tonsils are very enlarged, with a lot of exudates and the patient’s having a high temperature, I would still look at the blood test, [to] help give me an idea. If the blood test suggests a bacterial infection, I will cover the patient with antibiotics. If it doesn’t then I won’t…yeah.” – Participant 1 |
| 1. “If they insist I would usually give it to them but tell not to take it first, and to like…like you know keep it on standby, yeah. In case the fever persists for like another few days that kind of thing. I think they need it as a safety net in a way but I would definitely be explaining to them that most likely it’s still a viral infection and antibiotics don’t treat viruses. So there’s not much point anyway. But if they still insist, then I’ll tell them to take it on standby and only if they need it. Then at least they have that safety net.” – Participant 1 |
| 1. “I mean I’ve seen some cases where you know the consult is tricky, complex. It’s not so much because of the clinical part but there’s a lot of social problems…when there’s a lot of pressure and a lot of difficulty to end that consultation. So I’ve seen some cases where people prescribe a standby supply of antibiotics but telling the patient when may be a possible reason to take the antibiotics.” – Participant 4 |
| 1. “Even sometimes seniors they do, I’ve seen la, giving a standby supply of antibiotics. A one-week course, but maybe at that point the indication is still not strong and we still give the patients on discharge, maybe you can consider taking, if you know your fever is very persistent, if you have poor access to care, you’re feeling breathless, you may consider taking for a trial, watch for forty-eight hours, if not better, please come back, that kind of thing. There are some cases like that. Yeah but majority no.” – Participant 4 |
| 1. “I don’t think people would interfere with your management of an URTI. I think they sort of try, if you’re trying to bring it up…there may be something they want to learn also because obviously it’s not just the normal run of the mill cases.” – Participant 2 |
| 1. “If it’s not in alignment then I have to re-examine my position because if you’re saying peers, there’s a few other people who disagree with me then I have to re-examine my position right? Make sure that I’m not making a mistake. You see, so I would stand back and re-look at the situation from their point of view, you know what I mean? To see whether I am making a mistake.” – Participant 6 |
| 1. “I don’t know whether this is backed by evidence. Anecdotally, I find that a higher proportion of junior doctors feel less confident about not prescribing antibiotics. I’ve also observed some of the resident physicians who I would classify as more experienced physicians giving antibiotics more readily than the consultants or registrars.” – Participant 4 |
| **Hospital policies** |
| 1. “I always find that when you go to the outside general practitioners, they do this thing where they give one medicine for one symptom. Fever…one medicine, cough…one medicine. So when I start giving only two or three medicines in the ED, the patients are like why? I say this is two-in-one medication, don’t worry. This one medication is a two-in-one. This one stops phlegm, this one stops runny nose.” – Participant 6 |
| 1. “So yeah…my culture is very much this hospital’s culture. The culture here is the bottom line...is the cost, the cost to the patient. So it’s always like that you know, the treatments are based on that sort of principle. Don’t make them pay unnecessarily you know but at the same time, we are very scientific in how we approach it you see. But if you’re talking about resistance in the community, you need to address the polyclinics and the general practitioners, outside general practitioners. You should go interview some outside general practitioners because those people are autonomous. And they’re doing their own business and nobody dare to audit them except themselves. For them it’s running a business.” – Participant 6 |
| 1. “I think the locum sector in general practitioner settings need to be further policed, not just in URTI management. They could have done a better job in the primary care but they didn’t put extra effort or even enough effort. Then you just have to deal with it. Yeah because it is not uncommon you see in this referral letter that patient was given antibiotics…not getting better, so please review. Then after that you realise yah it’s still URTI, nothing special…or particular, then why did you refer the patient to me”? – Participant 4 |
| 1. “I wouldn’t think that hospital policy would help me. In fact, it is a shackle because it prevents me from exercising my own judgment. I know some people may consider that as a hospital policy or departmental policy, so as not to give [antibiotics] for the first presentation or whatever. I think that is more problematic than useful, so I don’t like that.” – Participant 4 |
| **Treatment guidelines** |
| 1. “It’s a combination of being taught by seniors, reading guidelines, there are actually a lot of guidelines on this like, the AFP, the American Association of Family Physicians…there’s the published, like those CENTORS guidelines that kind of thing for Strep throat. Clinical practice guidelines…CPGs. There in fact there was one CPG on URTI and I think rhino-sinusitis, recently I think, past few years... So all these help but of course, they are all guidelines and you make your own decision. Because they are not protocols. Guidelines are for consideration and your own adoption at the end. And all of them are actually very often low evidenced based, meaning they are not like randomised controlled trials…that kind of thing.” – Participant 4 |
| 1. “Usually URTI… they don’t have a guideline. Because it’s very broad one and then diagnosis is quite big. And then you cannot… if you like… want to see if pneumonia, there is guidelines we use over here for pneumonia called PSI, pulmonary severe index. Or you can try CURB-65. But over here, we don’t use CURB-65.” – Participant 5 |
| 1. “Usually when you start working here, the intranet [hospital’s internal website] will provide you with access to “UpToDate” and usually you can search for everything inside. So you will get the latest trends on what they recommend, what their randomised trials are showing, what were the previous results, what would be the percentage and what would be the wrong approach or wrong management. And if you want to know further details, you can click their link. It is useful, but you still need to apply your clinical judgment obviously. You cannot say “Oh okay…the UpToDate website is like this…so I will just treat all my patients this way. Every patient is unique and it may not be the same as what is on the textbook or whatever.” – Participant 5 |
| 1. “I started practicing way back in the year 2000…way back home. So we are, you are governed by all the… you know guidelines and teachings, so you go from there. But as you go on and see more patients, sometimes you can bend…you bend your rules. And how you have experienced this patient from one patient to another. But overall, you still have guidelines, which you have actually already used, which you may have practised…” – Participant 9 |
| 1. “ARUS-C system in this hospital is a very good tool… It’s easy to use. Having the same ARUS-C in the ED for I mean for… URTI then should be a good tool or guideline.” – Participant 8 |
| 1. “There is actually all those in ARUS-C system. What is this ARUS? So…that is the system which tells you which antibiotics to give as first-line for whatever [conditions]. As long as there is a guideline…if it’s a very clear-cut guideline and it’s not those which is neither here nor there…yes, that is very…very useful for me.” – Participant 9 |
| ***Community level*** |
| **Patient education and awareness** |
| 1. “My only message to you is education…and educate the doctors about antibiotic prescribing for URTI. That’s all, that’s all there is to it. You educate, you train one whole generation you know and set up a system to train the next and the next, that’s it.” – Participant 6 |
| 1. “I won’t…I would just have to educate them; I would have to. You know the treatment of URTI usually goes boils down to education…that’s the most important thing. The patients won’t understand. It’s not just simply giving antibiotics. You’ve to tell them I give you this antibiotics, later on you develop resistance. This is the first thing you have to tell them. You think you’ve got a fever right now, cough…sometimes viral fever can be up to five days. So I tell them it’s usually three to five days. Not unless you develop like difficulty breathing, like really… really, really bad…” – Participant 9 |
| 1. “Because it’s no point giving them something that they don’t know what it is, they don’t know what it’s for, and they just think oh every time the doctor just gives it to me. So why is the doctor not giving me now… so the patients don’t understand. I suppose if you explain to them then some of their unrealistic demands may then you know…be due to lack of understanding.” – Participant 2 |
| 1. “Because… I mean most clinicians are quite aware of the problem of antibiotic resistance. But the public in my opinion quite ignorant about this. I mean you look at the health seeking habits of Singaporeans. It is really like you know…for URTI, they will see one doctor. If not better, they go to another doctor. You know…they “doctor shop”. So I mean continuity of care is one issue. The other thing also is antibiotic resistance. The clinicians may not know what the previous doctor prescribed. If they prescribed something else, then… you know for one URTI they can receive two or three courses of antibiotics… which is really…really bad. So I think a lot of these issues require public education.” – Participant 7 |
| 1. “Education. We need to educate the people…we need to educate the doctors and the public. But public education…it’s more related to the fact that the general practitioner clinics and their individual doctors need to educate the patients at that point in time.” – Participant 6 |
